# Effects of Antiarrhythmic Drugs on hERG Gating in Human-Induced Pluripotent Stem Cell-Derived Cardiomyocytes From a Patient With Short QT Syndrome Type 1

**DOI:** 10.3389/fphar.2021.675003

**Published:** 2021-05-07

**Authors:** Mengying Huang, Zhenxing Liao, Xin Li, Zhen Yang, Xuehui Fan, Yingrui Li, Zhihan Zhao, Siegfried Lang, Lukas Cyganek, Xiaobo Zhou, Ibrahim Akin, Martin Borggrefe, Ibrahim El-Battrawy

**Affiliations:** ^1^ First Department of Medicine, Faculty of Medicine, University Medical Centre Mannheim (UMM), University of Heidelberg, Mannheim, Germany; ^2^ North Sichuan Medical College, Nanchong, China; ^3^ College of Medical Technology, Chengdu University of Traditional Chinese Medicine, Chengdu, China; ^4^ Key Laboratory of Medical Electrophysiology of Ministry of Education and Medical Electrophysiological Key Laboratory of Sichuan Province, Institute of Cardiovascular Research, Southwest Medical University, Luzhou, China; ^5^ DZHK (German Center for Cardiovascular Research), Partner Sites, Heidelberg-Mannheim and Göttingen, Mannheim, Germany; ^6^ Stem Cell Unit, Clinic for Cardiology and Pneumology, University Medical Center Göttingen, Göttingen, Germany

**Keywords:** short QT syndrome, arrhythmias, antiarrhythmic drugs, human-induced pluripotent stem cell-derived cardiomyocytes, hERG channel

## Abstract

**Aims:** The short QT syndrome type 1 (SQT1) is linked to hERG channel mutations (e.g., N588K). Drug effects on hERG channel gating kinetics in SQT1-cells have not been investigated.

**Methods:** This study used hiPSC-CMs of a healthy donor and a SQT1-patient carrying the N588K mutation and patch clamp to examine the drug effects on hERG channel gating kinetics.

**Results:** Ajmaline, amiodarone, ivabradine, flecainide, quinidine, mexiletine and ranolazine inhibited the hERG channel current (I_Kr_) less strongly in hiPSC-CMs from the SQTS1-patient (SQT1-hiPSC-CMs) comparing with cells from the healthy donor (donor-hiPSC-CMs). Quinidine and mexiletine reduced, but ajmaline, amiodarone, ivabradine and ranolazine increased the time to peak of I_Kr_ similarly in SQT1-hiPSC-CMs and donor-hiPSC-CMs. Although regarding the shift of activation and inactivation curves, tested drugs showed differential effects in donor- and SQT1-hiPSC-CMs, quinidine, ajmaline, ivabradine and mexiletine but not amiodarone, flecainide and ranolazine reduced the window current in SQT1-hiPSC-CMs. Quinidine, ajmaline, ivabradine and mexiletine differentially changed the time constant of recovery from inactivation, but all of them increased the time constant of deactivation in SQT1-hiPSC-CMs.

**Conclusion:** The window current-reducing and deactivation-slowing effects may be important for the antiarrhythmic effect of ajmaline, ivabradine, quinidine and mexiletine in SQT1-cells. This information may be helpful for selecting drugs for treating SQT1-patients with hERG channel mutation.

## Introduction

Short QT syndrome (SQTS), described initially by Gussak et al., in 2000 (Gussak et al., 2000), is a rare, inheritable heart disease associated with abbreviated corrected QT interval (QTc) and sudden cardiac death (SCD). So far, genes reported to be associated with SQTS include KCNH2 (SQTS1), KCNQ1 (SQTS2) and KCNJ2 (SQTS3) (Campuzano et al., 2018). In addition, a mutation in the cardiac Cl/HCO3 exchanger AE3 has also been reported in patients with SQTS (Thorsen et al., 2017).

The therapeutic approaches for SQTS are challenging for physicians because of the low prevalence and rare cases. Until now, a small number of drugs including disopyramide, nifekalant, quinidine, flecainide, sotalol, ibutilide and propafenone have been tested by *in vivo* studies on SQTS1 (Abriel and Rougier, 2013), among which only quinidine has shown profit effect in the treatment (Mizobuchi et al., 2008; Giustetto et al., 2011; Mazzanti et al., 2017; El-Battrawy et al., 2018; El-Battrawy et al., 2019). In human-induced stem cell-derived cardiomyocytes (hiPSC-CMs) from a patient with SQTS type 1, besides quinidine, disopyramide, ajmaline, ivabradine and mexiletine but not sotalol, amiodarone, flecainide and ranolazine showed profitable (APD-prolonging and antiarrhythmic) effects (Shinnawi et al., 2019; Zhao et al., 2019; Lan et al., 2020).

Since SQT1 is caused by an enhanced (a gain-of-function) hERG channel current, hERG channel blockers should be effective for prolonging QT interval or reducing the chance of arrhythmias in SQT1-patients. Surprisingly, some classical hERG channel blockers like sotalol and ibutilide failed to prolong QTc interval in SQT1-patients (Gaita et al., 2004; El-Battrawy et al., 2019), suggesting that the mutation in the hERG channel changed the sensitivity of the channel to drugs. Indeed, it was found that some drugs mainly affect hERG channels in the inactivated state and the SQTS1 is caused by a mutation in the hERG channel, which impairs inactivation of the channel and hence reduce the channel sensitivity to those drugs (McPate et al., 2008). Quinidine affects both the open and inactivated hERG channels and can still inhibit hERG channels even when the inactivation is impaired. This could be an explanation for the failure of some drugs in treating SQTS1. Therefore, numerous studies focused on influences of mutations on channel sensitivity and/or affinity of hERG channels to drugs. However, the sensitivity change cannot explain some phenomena, for example, amiodarone, propafenone and quinidine inhibited N588K-hERG channels in a similar intensity, but only quinidine is effective in prolonging QTc or action potential duration (APD) and suppressing arrhythmic events in SQT1-patients or SQT1-cells (McPate et al., 2008; Zhao et al., 2019). This suggests other mechanisms may also play important roles for the efficacy of drugs for SQTS-patients. In our recent studies, using hiPSC-CMs from a patient carrying N588K mutation in hERG channels, we tested the APD-prolonging and antiarrhythmic effects of quinidine, sotalol, ajmaline, amiodarone, ivabradine, flecainide, mexiletine and ranolazine, which are known to be able to inhibit hERG channels. We found that quinidine, ajmaline, ivabradine and mexiletine but not sotalol, amiodarone, flecainide and ranolazine prolonged APD and reduced arrhythmic events in SQT1-hiPSC-CMs (El-Battrawy et al., 2018; Zhao et al., 2019), although all of them could inhibit hERG channel currents. Therefore, we hypothesize that not only the channel sensitivity bot also the channel gating kinetics in presence of a drug is important for the efficacy of the drug and the observed differential drug effects may also result from different effects on hERG channel gating kinetics in SQT1-cells. The current study was designed to analyze in detail the effects of quinidine, ajmaline, amiodarone, ivabradine, flecainide, mexiletine and ranolazine on hERG channel gating kinetics in SQT1-hiPSC-CMs.

## Methods

### Ethics Statement and Clinical Data

A skin biopsy from a SQTS1 patient was obtained with written informed consent from the patient and the Ethics Committee of the Medical Faculty Mannheim, University of Heidelberg (approval numbers: 2018-565N-MA) and the Ethics Committee of University Medical Center Göttingen (approval number: 10/9/15). The study was carried out in accordance with the Helsinki Declaration of 1975 (https://www.wma.net/what-we-do/medical-ethics/declaration-of-helsinki/), revised in 2013.

### Generation of Human iPS Cells

The patient with familial SQTS1 carries the N588K mutation in hERG channel. The clinical data of the patient has been provided in our recent publication (El-Battrawy et al., 2018).

The methods for the generation of iPS cells (hiPSCs) have been described in our previous study (El-Battrawy et al., 2018). Briefly, skin fibroblasts from a skin biopsy of the patient and a healthy subject (here defined as donor) were reprogrammed into hiPSC cell line in feeder free culture conditions using the integration-free CytoTune-iPS 2.0 Sendai Reprogramming Kit (Thermo Fisher Scientific, #A16517) and the reprogramming factors OCT4, KLF4, SOX2 and c-MYC. The pluripotency and *in vitro* differentiation potential of generated hiPSCs were examined as described before (El-Battrawy et al., 2018). The detailed information is provided in the Supplementary Material.

### Generation of hiPSC-CMs

The hiPSCs were differentiated into cardiomyocytes (hiPSC-CMs) as described in our previous studies (Cyganek et al., 2018; El-Battrawy et al., 2018; El-Battrawy et al., 2018). Briefly (the detailed information is provided in the Supplementary Material), culture dishes were coated with Matrigel (Corning). The medium of TeSR-E8 (Stemcell Technologies) was used for hiPSCs culture and the medium of RPMI1640 Glutamax (Life Technologies) consisting of 1% sodium pyruvate, 1% Penicillin/Streptomycin, ascorbic acid (Sigma Aldrich) and B27 (Life Technologies) was used for hiPSC-CM culture (basic cardiac medium). In the first two weeks, CHIR99021 (Stemgent), BMP-4 (R&DSystems), FGF-2 (MiltenyiBiotec), Activin A (R&D Systems), and IWP-4 (Stemgent) were applied to induce hiPSCs to differentiate into hiPSC-CMs. Normally, on day 8 of differentiation some cells start to beat. In the third week, a selection medium containing sodium lactate (Sigma, Germany) and RPMI medium without glucose and glutamine (WKS, Germany) was used to select cardiomyocytes. Afterward, the selected cells were cultured with basic cardiac medium. After 40 to 60 days of differentiation, the cardiomyocytes were dissociated from 6 well plates and plated on Matrigel-coated 3.5 cm petri dishes as single cells for patch clamp tests. In our lab, the differentiation of hiPS cells into iPSC-CMs is regularly carried out every 2–3 weeks. The hiPSC-CMs from several differentiations were used for studies and the data were combined. Two clones of the hiPSCs were alternately differentiated into hiPSC-CMs.

### Patch-Clamp

Standard patch-clamp whole-cell recording techniques were used to measure the hERG channel currents at room temperature. Patch electrodes were pulled from borosilicate glass capillaries (MTW 150F; world Precision Instruments, Inc., Sarasota, FL) using a DMZ-Universal Puller (Zeitz-Instrumente Vertriebs GmbH, Martinsried, Germany) and filled with pre-filtered pipette solution (see below). Pipette resistance ranged from 1–2 MΩ. Signals were acquired at 10 kHz and filtered at 2 kHz with the EPC10 Patch-master digitizer hardware (HEKA Germany) and Fit-master software (HEKA Germany).

To separate the hERG channel current from other currents, the Cs^+^ currents conducted by hERG channels (I_Kr_) were measured. External solution contains (mM): 140 CsCl, 2 MgCl2, 10 HEPES, 10 Glucose, pH = 7.4 (CsOH). Pipette solution: 140 CsCl, 2 MgCl2, 10 HEPES, 10 EGTA, pH=7.2 (CsOH).

### Drugs

Ivabradine, flecainide, amiodarone, mexiletine, quinidine, and ranolazine were from Sigma, ajmaline from MP Biomedicals. The tested concentrations were selected according to literatures and our previous studies in hiPSC-CMs (Wu et al., 2004; El-Battrawy et al., 2018; Salvage et al., 2018; Zhao et al., 2019). Our previous studies showed that 10 µM quinidine, 10 µM ivabradine, 30 µM ajmaline, 100 µM mexiletine, prolonged APD, 10 µM amiodarone inhibited I_Kr_ (El-Battrawy et al., 2018; Zhao et al., 2019). Ranolazine (5–30 µM) significantly reduced episodes of EADs and VT produced by ATX-II (Wu et al., 2004). Flecainide of 3–30 µM was shown to inhibit Na and K channel currents (Salvage et al., 2018). Therefore, the concentrations of 10 µM quinidine, 30 µM ajmaline, 10 µM amiodarone, 10 µM ivabradine, 30 µM flecainide, 100 µM mexiletine and 30 µM ranolazine were chosen for the study. Of note, all these drugs can affect other channel currents besides hERG channel currents (Supplementary Table 1).

### Statistical Analysis

Data are shown as mean ± SEM and were analyzed using InStat^©^ (GraphPad, San Diego, United States) and SigmaPlot 11.0 (Systat GmbH, Germany). The t-test was used for comparison between two independent groups. Paired t-test was used for comparisons of data in the same cells before and after application of a drug. One way ANOVA was used for comparison among multiple groups (more than two groups). *p* < 0.05 (two-tailed) was considered significant.

## Results

### Drug Effects on hERG Channel Currents in hiPSC-CMs

**FIGURE 1:**
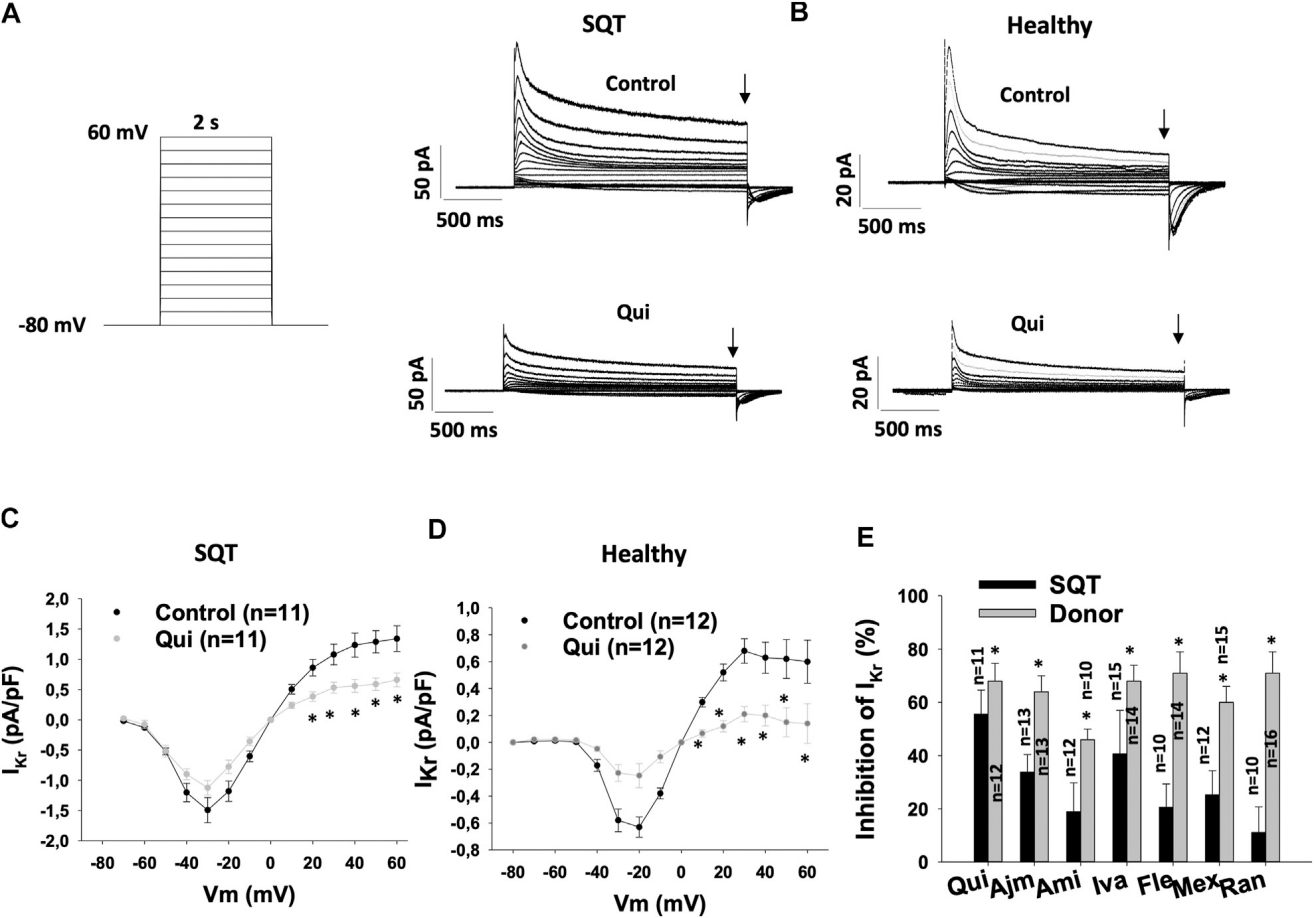
Drug effects on I_Kr_ in SQTS1-hiPSC-CMs. I_Kr_ was evoked by 2 s pulses from −80 to 60 mV (10 mV increments) with a holding potential of −80 mV. The steady state currents were measured at the end of pulses as indicated by arrows in **(A)** and **(B)** in the same cells before and after application of a drug. **(A)** The protocol **(right side)** and representative I_Kr_ traces **(left side)** in absence (Control) and presence of 10 µM quinidine (Qui) in a SQT1-hiPSC-CM (SQT). **(B)** Representative I_Kr_ traces in absence (Control) and presence of 10 µM quinidine (Qui) in a donor-hiPSC-CM (Healthy). **(C)** Current-voltage relationship (I-V) curves of I_Kr_ in absence (Control) and presence of 10 µM quinidine (Qui) in SQT1-hiPSC-CMs (SQT). **(D)** I-V curves of I_Kr_ in absence (Control) and presence of 10 µM quinidine (Qui) in donor-hiPSC-CMs (Healthy). **(E)** Averaged values of percent inhibition of I_Kr_ at 40 mV by quinidine (Qui, 10 µM), ajmaline (Ajm, 30 µM), amiodarone (Ami, 10 µM), ivabradine (Iva, 10 µM), flecainide (Fle, 30 µM), mexiletine (Mex, 100 µM) and ranolazine (Ran, 30 µM) in hiPSC-CMs from a healthy subject (Donor) and the patient (SQT). The inhibition was calculated by: Inhibition = (I_ctr_−I_drug_)/I_ctr_*100, where I_ctr_ is the current before application of a drug, I_drug_ is the steady state current in presence of a drug. Shown are mean ± SEM, *n* represents number of measured cells. The statistical significance was examined by paired t-test **(C, D)** or unpaired t-test **(E)**, *p < 0.05.

In our recent study, we demonstrated that quinidine, ajmaline, amiodarone, ivabradine, and mexiletine but not amiodarone, flecainide and ranolazine prolonged APD and reduced epinephrine induced arrhythmic events in hiPSC-CMs from the SQT1-patient (SQT1-hiPSC-CMs) (Zhao et al., 2019).To check whether these drugs affect hERG channel currents (I_Kr_) differentially in hiPSC-CMs from healthy donor and the SQTS1-patient, drug effects on I_Kr_ were analyzed in both the healthy and diseased cells. I_Kr_ amplitudes and current-voltage relationship (I-V) curves in the same cells before and after application of a drug were analyzed. Figures 1A–D showed effects of quinidine as examples of drug effects. Quinidine reduced the peak and steady state current of I_Kr_ (Figures 1,B) at different potentials (Figures 1C,D). All the tested seven drugs inhibited I_Kr_ in both donor- and SQT1-hiPSC-CMs. However, the inhibition induced by drugs was reduced in SQT1-hiPSC-CMs. Especially, the effect of amiodarone, flecainide, mexiletine and ranolazine was more severely reduced in SQT1-hiPSC-CMs (Figure 1E, Table 1).

**TABLE 1:** Summary of drug effects on I_Kr_ and APD/QT.

	Inhb (%)		T-P		Vh-act		τ-inact		Vh-inact		Win-cur		τ-rec		τ-deact		APD/QT-lit	
	SQT	H	SQT	H	SQT	H	SQT	H	SQT	H	SQT	H	SQT	H	SQT	H	SQT	H
Qui	55	68	−	−	R	R	N	+	L	L	−	−	+	+	+	+	P (Guo et al., 2019)	P (Davidenko et al., 1989)
Ajm	34	64	+	+	R	R	+	+	L	L	−	−	−	N	+	N	P (Zhao et al., 2019; Jæger et al., 2021)	P (Bébarová et al., 2005a)
Ami	19	46	+	+	N	R	−	+	N	R	N	N	−	−	N	−	N (Giustetto et al., 2011)	N (Aomine, 1988; Sasaki et al., 2014), P (Aomine, 1988)
Iva	41	68	+	+	R	R	−	−	L	R	−	N	N	+	+	+	P (Frommeyer et al., 2017)	N (Haechl et al., 2019), S (Koncz et al., 2011), P (Koncz et al., 2011)
Fle	21	71	N	+	N	N	+	+	N	L	N	N	+	N	N	N	N (Gaita et al., 2004)	P (Borchard and Boisten, 1982; Wang et al., 1993)
Mex	25	60	−−	−	N	R	N	−	L	L	−	−	−	N	+	+	P (Frommeyer et al., 2018)	S (Matsuo et al., 1985; Sato et al., 1995)
Ran	11	71	+	+	L	L	+	−	L	L	N	N	N	+	N	N	P (Frommeyer et al., 2016), N (Zhao et al., 2019)	P (Wu et al., 2004), S (Antzelevitch et al., 2004a)

I_Kr_: rapidly activating delay rectifier K (hERG) channel current. APD: action potential duration. SQT: SQT cells. H: healthy (wild-type) cells. Qui: quinidine. Ajm: ajmaline. Ami: amiodarone. Iva: ivabradine. Fle: flecainide. Mex: mexiletine. Ran: ranolazine. Inhb: inhibition induced by a drug. T-P: time to peak of I_Kr_. Vh-act: voltage at half maximal activation of I_Kr_. τ-inact: time constant of inactivation of I_Kr_. Vh-inact: voltage at half maximal inactivation of I_Kr_. Win-cur: window current of I_Kr_. τ-rec: time constant of recovery from inactivation of I_Kr_. APD/QT-lit: changes of APD or QT interval induced by the drug reported in literatures.

“−”: decrease. “+”: increase. N: No effect. R: Shift to a more positive potential (A shift to the right). L: Shift to a more negative potential (A shift to the left). P: Prolongation. S: shortening.

### Drug Effects on Activation of I_Kr_ in hiPSC-CMs

**FIGURE 2:**
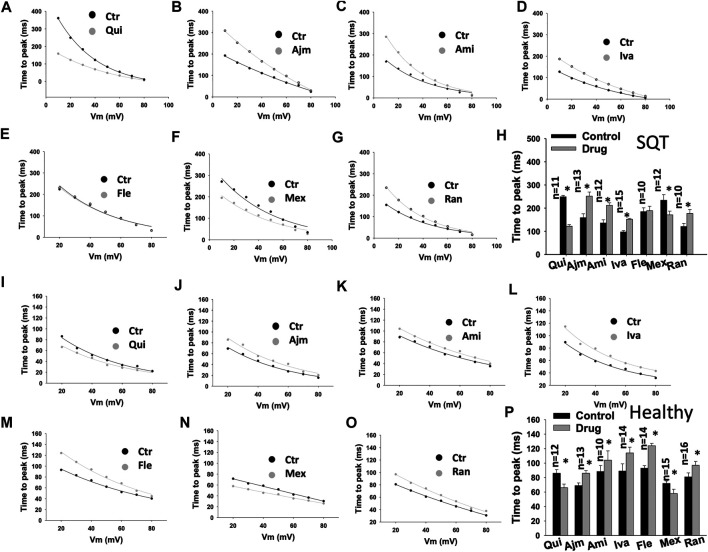
Drug effects on the time to peak of I_Kr_ in SQTS1-hiPSC-CMs. The time to peak (the time between the start of a pulse to the highest peak current point) of I_Kr_ was measured in current traces from 10 to 80 mV and plotted against voltages. The same cells before and after application of a drug were measured. **(A–G)** Representative curves of time to peak in SQT1-hiPSC-CMs in absence (Ctr) and presence of quinidine (Qui, 10 µM), ajmaline (Ajm, 30 µM), amiodarone (Ami, 10 µM), ivabradine (Iva, 10 µM), flecainide (Fle, 30 µM), mexiletine (Mex, 100 µM) and ranolazine (Ran, 30 µM). **(H)** Averaged values of time to peak at 20 mV in SQT1-hiPSC-CMs in absence (Ctr) and presence of drugs. **(I–O)** Representative curves of time to peak in donor-hiPSC-CMs in absence (Ctr) and presence of quinidine (Qui, 10 µM), ajmaline (Ajm, 30 µM), amiodarone (Ami, 10 µM), ivabradine (Iva, 10 µM), flecainide (Fle, 30 µM), mexiletine (Mex, 100 µM) and ranolazine (Ran, 30 µM). **(P)** Averaged values of time to peak at 20 mV in donor-hiPSC-CMs in absence (Ctr) and presence of drugs. Shown are mean ± SEM, *n* represents number of cells. The statistical significance was examined by paired t-test, *p < 0.05 versus Control.

To examine the effects of drugs on I_Kr_ activation, the time to peak (T-p) and activation curves were analyzed (Table 1). Quinidine (Figures 2A,H,I,P) and mexiletine (Figures 2F,H,N,P) accelerated (T-p was reduced), while ajmaline (Figures 2B,H,J,P), amiodarone (Figures 2C,H,K,P), ivabradine (Figures 2D,H,L,P) and ranolazine (Figures 2G,H,O,P) decelerated (T-p was increased) the I_Kr_ activation in donor- and SQTS1-hiPSC-CMs. Flecainide showed no effect in SQT1-hiPSC-CMs (Figures 2E,H), but increased T-p in donor-hiPSC-CMs (Figures 2M,P).

**FIGURE 3:**
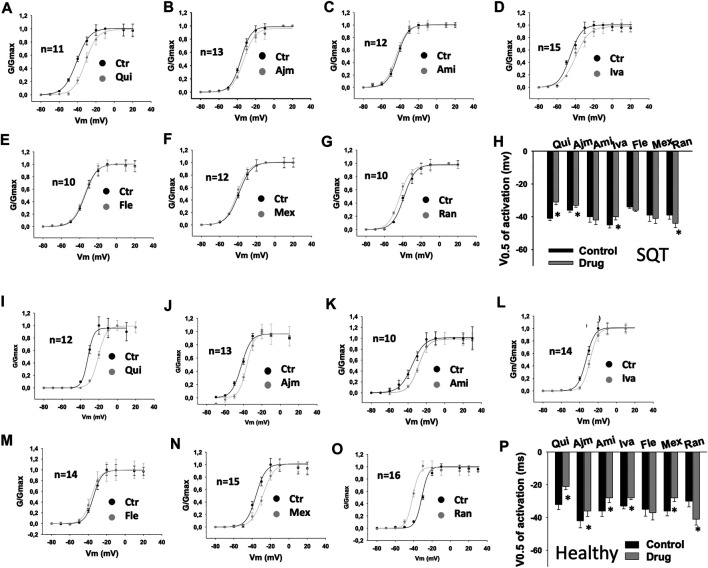
Drug effects on voltage-dependent activation of I_Kr_ in SQTS1-hiPSC-CMs. I_Kr_ was evoked by 2 s pulses from −80 to 60 mV (10 mV increments) with a holding potential of −80 mV. The steady state currents were measured at the end of pulses as indicated by arrows in Figures 1A,B before and after application of a drug. The conductance was calculated with the equation: G = I/V, where I is the measured current, V is the voltage of pulses and G is the conductance. The conductance was normalized to the maximum (G/Gmax) and plotted against voltage to obtain the activation (G-V) curves. The curves were fitted by Boltzmann equation to obtain the voltage value at half maximal activation (V0.5). **(A–G)** Activation curves of I_Kr_ in SQT1-hiPSC-CMs in absence (Ctr) and presence of quinidine (Qui, 10 µM), ajmaline (Ajm, 30 µM), amiodarone (Ami, 10 µM), ivabradine (Iva, 10 µM), flecainide (Fle, 30 µM), mexiletine (Mex, 100 µM) and ranolazine (Ran, 30 µM). **(H)** Averaged values of half maximal activation potential (V0.5) in SQT1-hiPSC-CMs in absence (Ctr) and presence of drugs. **(I–O)** Activation curves of I_Kr_ in donor-hiPSC-CMs in absence (Ctr) and presence of quinidine (Qui, 10 µM), ajmaline (Ajm, 30 µM), amiodarone (Ami, 10 µM), ivabradine (Iva, 10 µM), flecainide (Fle, 30 µM), mexiletine (Mex, 100 µM) and ranolazine (Ran, 30 µM). **(P)** Averaged values of half maximal activation potential (V0.5) in donor-hiPSC-CMs in absence (Ctr) and presence of drugs. Shown are mean ± SEM, *n* represents number of cells. The statistical significance was examined by paired t-test, *p < 0.05 versus Control.

In SQT1-hiPSC-CMs, quinidine, ajmaline and ivabradine shifted the activation curve of I_Kr_ to more positive potentials (Figures 3A,B,D,H), whereas ranolazine shifted the activation curve to more negative potentials (Figures 3G,H). Amiodarone, flecainide and mexiletine showed no effect (Figures 3C,E–H). In donor cells, quinidine, ajmaline, amiodarone, ivabradine and mexiletine shifted the activation curves to more positive potentials (Figures 3I–L,N,P), while ranolazine showed an opposite effect (Figures 3O,P). Flecainide showed no significant effect (Figures 3M,P).

### Drug Effects on Inactivation of I_Kr_ in hiPSC-CMs

**FIGURE 4:**
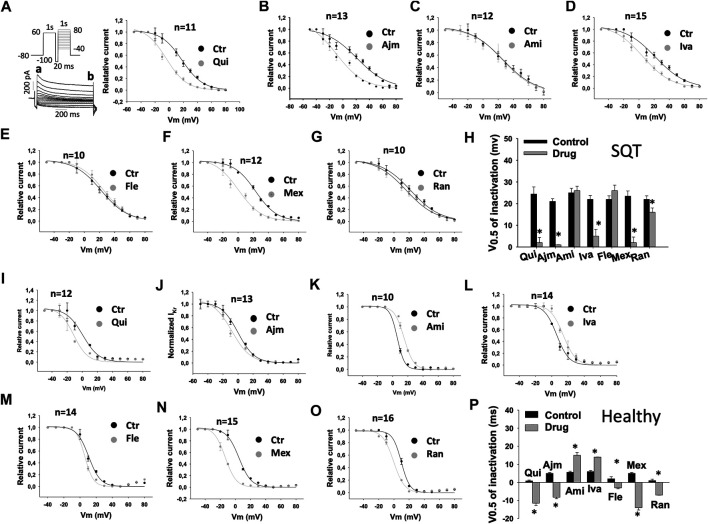
Drug effects on voltage-dependent inactivation of I_Kr_ in SQTS1-hiPSC-CMs. I_Kr_ was evoked by the protocol shown in A **(right panel)**. A pre-pulse of 1 s from −80 mV (the holding potential) to +60 mV followed by a short (20 ms) repolarization to −100 mV was given for channel activation, inactivation and recovery of inactivated channels to open state. Before deactivation occurs, test pulses of 1°s from −50 to +80 mV (10 mV increments) were started to evaluate the inactivation of channels. I_Kr_ was measured at the beginning (peak current, a) and end (steady current, b) of test pulses as shown in A **(right panel)**. The currents in same cells before and after application of a drug were measured. The current of inactivated channels (I_inact_) was calculated as: I_inact_ =Ia-Ib, where Ia is the peak current, Ib is the steady current. When I_inact_ is larger than zero, it means inactivation occurred. When I_inact_ equals or is smaller than zero, it means no inactivation occurred. I_inact_ at different potentials were measured and normalized to the maximal current (I_inactMax_). To plot the inactivation curves as usually shown (the curve decays with increasing voltages), the relative current was calculated by I=1 − I_inact_/I_inactMax_. Finally, I was normalized to the maximal value I_max_ and plotted against voltages to obtain an inactivation curve. The inactivation curves were then fitted by Boltzmann equation to obtain the voltage value at half maximal inactivation (V0.5). **(A–G)** Inactivation curves of I_Kr_ in SQT1-hiPSC-CMs in absence (Ctr) and presence of quinidine (Qui, 10 µM), ajmaline (Ajm, 30 µM), amiodarone (Ami, 10 µM), ivabradine (Iva, 10 µM), flecainide (Fle, 30 µM), mexiletine (Mex, 100 µM) and ranolazine (Ran, 30 µM). **(H)** Averaged values of half maximal inactivation potential (V0.5) in SQT1-hiPSC-CMs in absence (Ctr) and presence of drugs. **(I–O)** Inactivation curves of I_Kr_ in donor-hiPSC-CMs in absence (Ctr) and presence of quinidine (Qui, 10 µM), ajmaline (Ajm, 30 µM), amiodarone (Ami, 10 µM), ivabradine (Iva, 10 µM), flecainide (Fle, 30 µM), mexiletine (Mex, 100 µM) and ranolazine (Ran, 30 µM). **(P)** Averaged values of half maximal inactivation potential (V0.5) in donor-hiPSC-CMs in absence (Ctr) and presence of drugs. Shown are mean ± SEM, *n* represents number of cells. The statistical significance was examined by paired t-test, *p < 0.05 versus Control.

To examine drug effects on I_Kr_ inactivation, the inactivation curves of I_Kr_ and the time constant (τ) of the current decay due to channel inactivation were analyzed (Table 1). The inactivation curves were shifted to more negative potentials by quinidine (Figures 4A,H), ajmaline (Figures 4B,H), ivabradine (Figures 4D,H), mexiletine (Figures 4F,H), and ranolazine (Figures 4G,H) but not significantly influenced by amiodarone (Figures 4C,H) and flecainide (Figures 4E,H) in SQT1-hiPSC-CMs. In donor cells, the inactivation curves were shifted to more negative potentials by quinidine (Figures 4I,P), ajmaline (Figures 4J,P), flecainide (Figures 4M,P), mexiletine (Figures 4N,P) and ranolazine (Figures 4O,P) and to more positive potentials by amiodarone (Figures 4K,P) and ivabradine (Figures 4L,P).

**FIGURE 5:**
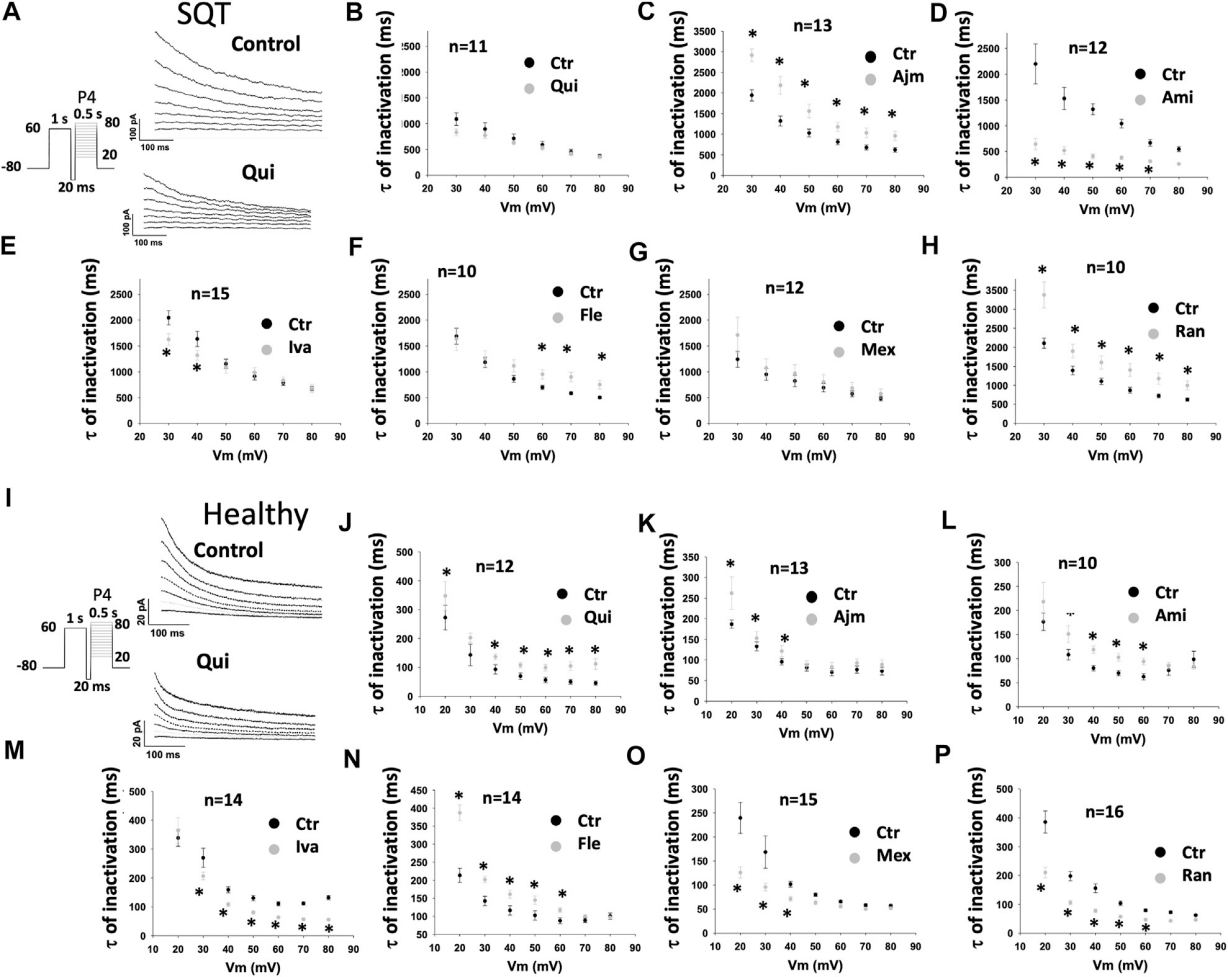
Drug effects on the time constant of I_Kr_ inactivation in SQTS1-hiPSC-CMs. To examine drug effects on the fast phase of inactivation, I_Kr_ was evoked by a 1 s-long pre-pulse from −80 to 60 mV to let hERG channels be activated and returned to −100 mV for 20 ms, and then the pre-pulse was followed by 500 ms test-pulses [P4 in the protocol shown by the inset in **(A)** and **(I)**] from 20 to 80 mV (10 mV increments). Currents evoked by test pulses were fitted by single exponential decay to obtain the time constant (τ) of I_Kr_ fast inactivation. **(A)** Representative traces of I_Kr_ evoked by pulses from 20 to 80 mV in absence (Control) and presence of 10 µM quinidine (Qui) in a SQT1-hiPSC-CM. **(B–H)** Mean values of time constants (τ) of I_Kr_ inactivation in SQT1-hiPSC-CMs in absence (Ctr) and presence of quinidine (Qui, 10 µM), ajmaline (Ajm, 30 µM), amiodarone (Ami, 10 µM), ivabradine (Iva, 10 µM), flecainide (Fle, 30 µM), mexiletine (Mex, 100 µM) and ranolazine (Ran, 30 µM). **(I)** Representative traces of I_Kr_ evoked by pulses from 20 to 80 mV in absence (Control) and presence of 10 µM quinidine (Qui) in a donor-hiPSC-CM. **(J–P)** Mean values of time constants (τ) of I_Kr_ inactivation in donor-hiPSC-CMs in absence (Ctr) and presence of each drug. Shown are mean ± SEM, *n* represents number of cells. The statistical significance was examined by paired t-test, *p < 0.05 versus Control.

In addition, it was detected that in SQT1-hiPSC-CMs, ajmaline (Figure 5C), flecainide (Figure 5F) and ranolazine (Figure 5H) decelerated the inactivation (time constant was increased), while amiodarone (Figure 5D) and ivabradine (Figure 5E) accelerated it. Quinidine (Figure 5B) and mexiletine (Figure 5G) had no significant effects. In donor-hiPSC-CMs, quinidine (Figure 5J), ajmaline (Figure 5K), amiodarone (Figure 5L) and flecainide (Figure 5N) decelerated the inactivation, while ivabradine (Figure 5M), mexiletine (Figure 5O) and ranolazine (Figure 5P) accelerated it. Figures 5A,I show examples of current inactivation in absence and presence of a drug (quinidine).

### Drug Effects on I_Kr_ Window Currents in hiPSC-CMs

**FIGURE 6:**
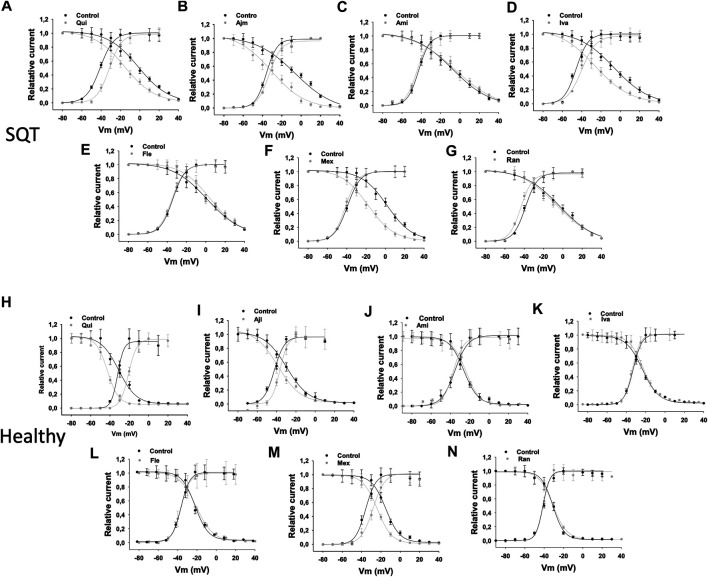
Drug effects on window currents of I_Kr_ in SQTS1-hiPSC-CMs. The activation and inactivation curves were plotted in the same figure. The window current was defined as the current under the crossover of activation and inactivation curves. The activation curves are the same curves as in Figure 3. To obtain the inactivation curves, a pulse of 1 s from −80 to +60 mV was applied to activate and inactivate hERG channels. Then, the pulse was set to −100 mV for 20 ms to let the inactivated channels recover to open state. Before the deactivation occurs, a test pulse to +60 mV for 500 ms was applied and the peak current at the test potential was measured. The voltage of recovery pulses was changed from −100 to +80 mV. The currents at test pulse versus respective voltages of the recovery pulses were normalized to the maximal current and plotted against voltages of the recovery pulses to obtain the inactivation curves (availability-voltage curves). The cell numbers of activation curves are same as that in Figure 3. The cells numbers of inactivation curves are nine for each drug experiments. For comparison, the window currents in absence (black points and lines) and presence (grey points and lines) of drugs were overlapped in the same plot. **(A–G)** Overlapped activation and inactivation curves showing the widow currents in SQT1-hiPSC-CMs in absence (Ctr) and presence of quinidine (Qui, 10 µM), ajmaline (Ajm, 30 µM), amiodarone (Ami, 10 µM), ivabradine (Iva, 10 µM), flecainide (Fle, 30 µM), mexiletine (Mex, 100 µM) and ranolazine (Ran, 30 µM). **(H–N)** Overlapped activation and inactivation curves showing the widow currents in donor-hiPSC-CMs in absence (Ctr) and presence of each drug.

Since the shift of activation or inactivation curves may change the window current, we assessed the I_Kr_ window current in the same cells in absence and presence of a drug. Quinidine, ivabradine, ajmaline and mexiletine reduced the I_Kr_ window current, but amiodarone, flecainide and ranolazine failed to do so in SQTS1-hiPSC-CMs (Figures 6A–G). In donor cells, quinidine, ajmaline and mexiletine also reduced the window current, whereas ivabradine, ranolazine, amiodarone and flecainide showed no effects (Figures 6 H–N) (Table 1).

### Drug Effects on Recovery From Inactivation of I_Kr_ in hiPSC-CMs

**FIGURE 7:**
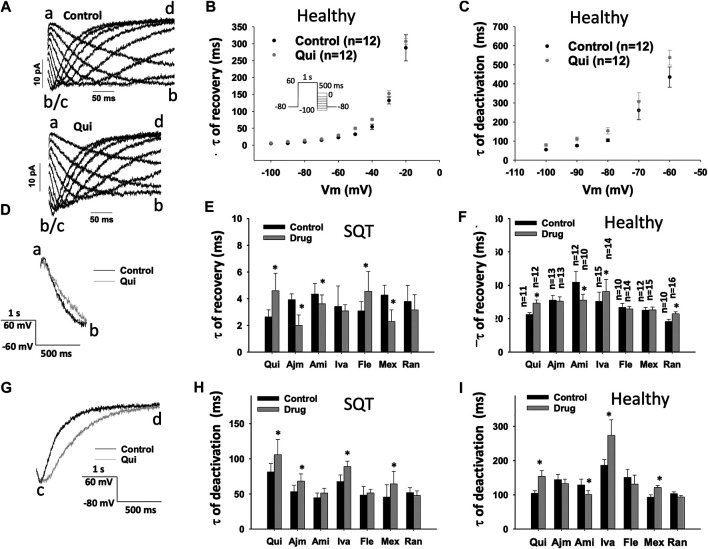
Drug effects on time constants of I_Kr_ recovery from inactivation and deactivation in SQTS1-hiPSC-CMs. I_Kr_ was evoked by a 1 s-long pre-pulse from −80 to 60 mV to let hERG channels be activated and some of them to be inactivated. The pre-pulse was followed by 500 ms test-pulses from −20 to −100 mV (10 mV increments). The protocol is shown in **(B)** (inset). Currents evoked by test pulses (tail currents) were used for analyzing the recovery from inactivation or deactivation of the currents. When the tail current increased, i.e., “b” is larger (more negative) than “a”, the current was defined as recovered current. The curves were fitted by single exponential decay to obtain time constants of recovery from inactivation. From −50 to −100 mV, deactivation became obvious. i.e., the current decrease again (the current at “d” is smaller than that at “c”. The change point “b/c” is defined as the end of recovered current and the start of the deactivation. **(A)** Representative traces of I_Kr_ evoked by test pulses from −20 to −100 mV in absence (Control) and presence of 10 µM quinidine (Qui) in a donor-hiPSC-CM. **(B)** Mean values of time constants (τ) of I_Kr_ recovery from inactivation at different voltages in donor-hiPSC-CMs in absence (Ctr) and presence of quinidine. **(C)** Mean values of time constants (τ) of I_Kr_ deactivation at different voltages in donor-hiPSC-CMs in absence (Ctr) and presence of quinidine. **(D)** Representative traces of I_Kr_ at −60 mV taken as recovered currents from inactivation in absence (Control) and presence of 10 µM quinidine (Qui) in donor-hiPSC-CMs. **(E)** Mean values of time constants (τ) of I_Kr_ recovery from inactivation at −60 mV in SQT1-hiPSC-CMs in absence (Ctr) and presence of quinidine (Qui, 10 µM), ajmaline (Ajm, 30 µM), amiodarone (Ami, 10 µM), ivabradine (Iva, 10 µM), flecainide (Fle, 30 µM), mexiletine (Mex, 100 µM) and ranolazine (Ran, 30 µM). **(F)** Mean values of time constants (τ) of I_Kr_ recovery from inactivation at −60 mV in donor-hiPSC-CMs in absence (Ctr) and presence of each drug. **(G)** Representative traces of I_Kr_ at −80 mV taken as deactivated currents in absence (Control) and presence of 10 µM quinidine (Qui) in a donor-hiPSC-CM. **(H)** Mean values of time constants (τ) of I_Kr_ deactivation at −80 mV in SQT1-hiPSC-CMs in absence (Ctr) and presence of quinidine (Qui, 10 µM), ajmaline (Ajm, 30 µM), amiodarone (Ami, 10 µM), ivabradine (Iva, 10 µM), flecainide (Fle, 30 µM), mexiletine (Mex, 100 µM) and ranolazine (Ran, 30 µM). **(I)** Mean values of time constants (τ) of I_Kr_ deactivation at −80 mV in donor-hiPSC-CMs in absence (Ctr) and presence of each drug. Shown are mean ± SEM, n represents number of cells. The n-numbers given in **(F)** are also for **(E)**, **(H)** and **(I)**. The statistical significance was examined by paired t-test, *p < 0.05 *vs.* Control.

To examine drug effect on the recovery of hERG channel from inactivation, the time constant (τ) of tail currents representing recovery of hERG channels from inactivation was analyzed (Figures 7E,F) (Table 1). In SQTS1-hiPSC-CMs, ajmaline, amiodarone and mexiletine speeded up the recovery, whereas flecainide and quinidine slowed it down. Ivabradine and ranolazine showed no significant effect (Figure 7E). In donor cells, the recovery speed was reduced by quinidine (Figures 7B,F), ivabradine and ranolazine, increased by amiodarone but not significantly affected by ajmaline, flecainide and mexiletine (Figure 7F). Figures 7A,D show examples of current recovery from inactivation in absence and presence of a drug (quinidine).

### Drug Effects on Deactivation of I_Kr_ in hiPSC-CMs

Finally, the deactivation of I_Kr_ was analyzed in hiPSC-CMs (Table 1, Figures 7A,G–I). In SQT1-hiPSC-CMs, quinidine, ajmaline, ivabradine and mexiletine reduced the speed of deactivation of hERG channels (the time constant τ was increased), but amiodarone, flecainide and ranolazine failed to change it (Figure 7H). In donor cells, amiodarone accelerated the deactivation (Figure 7I), while quinidine (Figures 7C,I), ivabradine and mexiletine decelerated it (Figure 7I). Ajmaline, flecainide and ranolazine did not influence the channel deactivation (Figure 7I). Figures 7A,G show examples of current deactivation in absence and presence of a drug (quinidine).

## Discussion

In this study, we investigated the effects of quinidine, ajmaline, amiodarone, ivabradine, flecainide, mexiletine and ranolazine on gating kinetics of hERG channels in hiPSC-CMs from a SQT1-patient with N588k mutation. The study demonstrated that 1) these drugs affected differentially the hERG channel gating kinetics in donor- and SQT1-hiPSC-CMs, 2) quinidine, ajmaline, ivabradine and mexiletine reduced the window current of hERG channels and 3) quinidine, ajmaline, ivabradine and mexiletine decelerated deactivation of hERG channels. The latter two may be important for their antiarrhythmic effects previously observed in SQTS-patients (reduction of the occurrence of life-threatening arrhythmic events) or SQTS-hiPSC-CMs (reduction of epinephrine-induced arrhythmic events) (Mazzanti et al., 2017; Zhao et al., 2019).

In our previous study, we generated hiPSC-CMs from a patient with STQS1 carrying the hERG (KCNH2) gene mutation of N588K and characterized the phenotypic features (APD-shortening and arrhythmic events) (El-Battrawy et al., 2018). Using the cellular model of SQT1 (SQT1-hiPSC-CMs) we tested the APD-prolonging and antiarrhythmic effect of different drugs (El-Battrawy et al., 2018; Zhao et al., 2019; Lan et al., 2020). We have demonstrated that the SQTS1-hiPSC-CMs displayed shortened APD and increased arrhythmic events. Quinidine, disopyramide, ajmaline, ivabradine and mexiletine but not amiodarone, flecainide or ranolazine prolonged APD and reduced the arrhythmic episodes in SQT1-hiPSC-CMs (El-Battrawy et al., 2018; Zhao et al., 2019; Lan et al., 2020). Although we found that the inhibition of the N588K-hERG channel current by drugs is different, whether these drugs exert also different effects on N588K-hERG channel gating kinetics, which may help clarify the effect difference of drugs, is so far unknown. The current study was designed to address this open question and look for hints for selecting possible effective drugs for SQT1-treatment.

Quinidine is a multiple channel blocker with a high affinity to hERG and was a frequently used antiarrhythmic drug. It can inhibit I_Na_ (Na channel current), I_Ca-L_ (L-type Ca current), I_to_ (transient outward K current), I_Kr_ (hERG channel current), I_Ks_ (slowly activating delayed rectifier K current), I_K1_ (inward rectifier K current) and I_KATP_ (ATP-sensitive-K channel current) (Supplementary Table 1) (Pugsley et al., 2005; Michel et al., 2002; Paul et al., 2002; Yang et al., 2009; Imaizumi and Giles, 1987; Koepple et al., 2017; Undrovinas et al., 1990). The I_Na_-inhibiting effect can reduce the speed of depolarization and excitation conduction, and hence reduce occurrence of some arrhythmias. The inhibition of I_Ca-L_ may shorten APD, whereas the inhibition of K currents can prolong APD. In fact, quinidine prolongs APD in wild-type (healthy) and SQTS-cells (Table 1) (Davidenko et al., 1989; Guo et al., 2019), indicating that its effect on K currents is larger than that on I_Ca-L_. The APD-prolonging effect can be antiarrhythmic and also proarrhythmic. Quinidine was clinically used as an antiarrhythmic drug, but the severe side effects of quinidine reduced its clinical relevance for arrhythmia-treatment. However, when it was shown to be effective for SQT-patients, it absorbed again interests of physicians and researchers. It was found that although the mutation N588K in hERG channel rendered hERG channel resistant to many drugs including typical hERG channel blockers, the channel remains still sensitive to quinidine. One reason for the efficacy of quinidine is that quinidine affects hERG channels in both open and closed (inactivated) state, different from other drugs that affect only or mainly inactivated hERG channels. The N588K mutation reduced the inactivation of hERG channel and hence reduced the effects of inactivation-affecting drugs like E-4031, sotalol, dofetilide and terfenadine (McPate et al., 2008; Perrin et al., 2008). Quinidine effect is only partially changed by N588K mutation because its open-channel-affecting effect is not influenced by the mutation. Therefore, quinidine is effective for SQT-patients with N588K mutation and recommended for application in SQT-patients (Priori et al., 2015). Since previously reported studies focused on the change of channel sensitivity (shift of dose-response curve) induced by N588K-mutation, the influence of N588K on drug effects regarding the hERG channel gating kinetic parameters was not analyzed in detail. In the current study, we found out that the quinidine effects on the time to peak and activation curve, on the time constant of inactivation and inactivation curve, on the time constants of recovery from inactivation and the deactivation as well as on the window current of the hERG channel were similar in donor- and SQT1-hiPSC-CMs, suggesting that quinidine effects on hERG channel gating are not severely changed by N588K.

The class Ia anti-arrhythmic drug ajmaline is frequently applied to induce phenotypic changes of Brugada syndrome (BrS) in ECG for diagnostic aim. Studies reported that ajmaline inhibits various currents, including I_Na_, I_Ca-L_, I_to_, I_Kr_, I_K1_ and I_KATP_ (Supplementary Table 1) (Miller et al., 2017; Bébarová et al., 2005a; Khodorov and Zaborovskaya, 1983; Bébarová et al., 2005b; Kiesecker et al., 2004). The inhibition of I_to_, I_Kr_, I_K1_ and I_KATP_ can prolong APD and hence APD-prolongation induced by ajmaline was observed in both wild-type cells and an SQT-model (Table 1) (Bébarová et al., 2005b; Jæger et al., 2021). This led us to assume that it may inhibit the hERG channel current in SQTS1-hiPSC-CMs. Indeed, ajmaline inhibited the hERG channel current, prolonged APD and reduced arrhythmic events in SQTS1-cells (Zhao et al., 2019). In HEK cells and Xenopus oocytes expressing hERG channels, ajmaline inhibited the hERG current and the Y652A and F656A mutations abolished the ajmaline effect (Kiesecker et al., 2004). Ajmaline shifted slightly the half-maximal activation voltage toward more negative potentials but did not significantly influence hERG inactivation curve, although it reduced the τ value of inactivation (Kiesecker et al., 2004). The influence of N588K on ajmaline effects was not examined in that study. Here in the current study, we add novel data about ajmaline effects on the gating parameters of wild-type and N588K-hERG channels in hiPSC-CMs. The effects of ajmaline on hERG channel current data including the time to peak, τ of inactivation, activation and inactivation curves and the window current are similar (changes in the same direction) in donor- and SQT1-hiPSC-CMs, while its effects on the time constants of recovery from inactivation and deactivation were observed only in SQT1-hiPSC-CMs. These data may be helpful for understanding the anti- or pro-arrhythmic feature of the drug. Since ajmaline can inhibit other ion channel currents, its inhibitory effect on I_to_, I_K1_ and I_KATP_ may also contribute its APD-prolonging and antiarrhythmic effects in SQTS.

Amiodarone alone showed no QTc-prolonging effect, but amiodarone together with metoprolol prolonged QTc in a SQTS-patient (Giustetto et al., 2011). The benefit of amiodarone for SQTS-patients is still questionable. In our hiPS-CMs from the patient with SQTS1, amiodarone failed to prolong APD and to reduce arrhythmic events. Amiodarone is similar to quinidine in inhibiting hERG current conducted by the mutant (N588K) hERG channel (McPate et al., 2008). Since the influence of N588K on amiodarone effects regarding gating kinetics of hERG channels was not investigated, it is difficult to understand why quinidine but not amiodarone is effective in SQT1-patients. Our current study detected the difference, i.e., quinidine reduced the window current, decelerated the recovery from inactivation and deactivation, while amiodarone showed no or opposite effects on those parameters. Besides hERG current, I_Na_, I_Ca-L_, I_NCX_, I_to_, I_Ks_, I_K1_ and I_KATP_ can be inhibited by amiodarone (Supplementary Table 1) (Nishida et al., 2011; Suzuki et al., 2013; Iwamoto et al., 2007; Watanabe, 2019). The inhibition of I_Ca-L_ may shorten APD, while the inhibition of I_to_, I_Ks_, I_K1_ and I_KATP_ can prolong APD. In wild-type cells, amiodarone prolonged or showed no effect on APD (Table 1) (Aomine, 1988; Sasaki et al., 2014). In our experiments, amiodarone failed to changed APD significantly, implying that its effects on inward currents and outward currents are similar.

Ivabradine could prolong QT interval and suppress the arrhythmic events in rabbit models of SQTS induced by an ATP-sensitive potassium channel activator pinacidil (Frommeyer et al., 2017). Ivabradine is a well-known funny channel (I_f_) blocker. Since it displayed no negative inotropic effects and is useful to maintain stable hemodynamics, it is clinically administered to reduce heart rate in patients with heart failure. Ivabradine also inhibits hERG channel currents (Lees-Miller et al., 2015). In a study, hERG channels, either WT or mutated, were expressed by transfection into HEK393 cells and ivabradine effects on the channels were investigated (Melgari et al., 2015). In that study, Melgari et al. demonstrated that ivabradine inhibited the WT- and mutated hERG channel current. N588K and S624A variants slightly reduced ivabradine effect, while Y652A and F656A strongly reduced its effect. In addition, the authors also showed that ivabradine shifted both the activation and inactivation curve to a more negative potential without effect on the speed (τ) of inactivation of the WT-hERG channel. In the current study, we demonstrated that the inhibition of hERG current by ivabradine was reduced and the inactivation curve was shifted to a more negative potential, consistent with the previous study. However, we found that ivabradine shifted the activation curve to a more positive potential, which differs from the results of the Melgari study. Moreover, we observed that ivabradine prolonged the time to peak, reduced the time constant of inactivation and increased the time constant of deactivation in both donor and SQT1-cells, reduced the window current in SQT1-cells but not in donor-cells, and increased the time constant of recovery in donor-cells but not in SQT1-cells. These data are new because previous studies including Melgari study did not investigate ivabradine effects on these parameters in N588K-hERG channels or in SQT1-cells. Besides hERG current, I_Na_, but not I_Ca-L_, I_Ks_ and I_K1_ can be inhibited by ivabradine (Supplementary Table 1) (Haechl et al., 2019; Demontis et al., 2009). The inhibition of I_Na_ may contribute to antiarrhythmic but not APD-prolonging effect. In previous studies, ivabradine showed no effect or prolonging or shortening effect on APD in wild type cells (Table 1) (Koncz et al., 2011; Haechl et al., 2019). It seems that ivabradine effect on APD mainly resulted from its effect on I_Kr_.

Mexiletine, a sodium channel blocker, was shown to be effective for suppressing arrhythmias in a rabbit model of SQTS and atrial fibrillation (AF) (Frommeyer et al., 2018). In our recent study, using the SQT1-hiPSC-CMs we also observed profitable results of mexiletine, prolonging APD and reducing arrhythmic events. Although mexiletine inhibited I_Kr_ in SQTS1-hiPSC-CMs, its effect on I_Kr_ gating kinetic parameter has not been assessed. In a previous study from Gualdani et al., WT and mutant hERG channels were expressed in HEK293 or CHO cells and mexiletine effects were assessed (Gualdani et al., 2015). The study demonstrated that i) Mexiletine inhibited the hERG channel current in a time- and voltage-dependent way; ii) Its inhibitory effect was strongly reduced by Y652A and F656A mutants; iii) It reduced the time constant of activation but increased the time constant of deactivation, meaning that it accelerated activation and decelerated deactivation; iv) Mexiletine did not influence the channel inactivation. The effects of mexiletine on gating kinetics of N588K-hERG channels were not investigated. Our current study detected that the inhibitory effect of mexiletine was strongly reduced in SQT1-hiPSC-CMs carrying the N588K mutation. Mexiletine reduced the time to peak in both donor and SQT1-cells, suggesting an increase in activation speed, consistent with the Gualdani study. In our study, mexiletine increased the time constant of deactivation (consistent with the previous study) but reduced the time constant of inactivation (different from the previous study) in both donor and SQT1-cells. The differences between our and the previous study may result from using different cell types. Further, mexiletine reduced the time constant of recovery from inactivation and the window current as well, which have not been shown before. In addition, mexiletine can inhibit I_Na_, I_Ca-L_, I_NCX_, I_Ks_ and I_to,_ and activate I_KATP_ (Supplementary Table 1) (Wang et al., 1996; Yonemizu et al., 2019; Rahm et al., 2020; Mitcheson and Hancox, 1997). The I_Ca-L_-inhibiting and I_KATP_-activation can explain the APD-shortening effect of mexiletine in wild-type cardiomyocytes (Table 1) (Matsuo et al., 1985; Sato et al., 1995). The I_Kr_-, I_Ks_- and I_to_-inhibiting effects can be the reason for APD-prolonging effect observed in SQT-cells (Frommeyer et al., 2018). Whether mexiletine prolongs or shortens APD in cells, probably depends on its net effect on the inward and outward currents. In our hiPSC-CMs, mexiletine prolonged APD, suggesting that its effect on outward current is predominant.

Flecainide has been shown to prolong APD and refractory period but did not reduce the rate of inducibility of ventricular fibrillation in a rabbit model of SQTS (Milberg et al., 2007). In a clinical study, it was shown to be ineffective for prolonging QT interval and reducing arrhythmias (Gaita et al., 2004). In our recent study, it inhibited slightly the hERG channel current, but failed to prolong APD or to reduce arrhythmic events in SQT1-hiPSC-CMs (Zhao et al., 2019). Before, it was shown that flecainide inhibited hERG channel currents in HEK cells and N588K reduced flecainide effect (Melgari et al., 2015). Flecainide did not influence the voltage-dependent inactivation curve but increased the time constant of inactivation of wild-type hERG channels (Melgari et al., 2015). Flecainide shifted activation curve to a more negative potential but did not influence the time constant of deactivation of wild-type hERG channels (Paul et al., 2002). Flecainide effects on gating kinetics of N588K-hERG channel have not been reported. Our current study showed that i) The inhibitory effect of flecainide was largely reduced in SQT1-cells, ii) The time to peak of hERG current was prolonged in donor but not in SQT1-cells and iii) The inactivation curve was shifted to a more negative potential in donor but not in SQT1-cells. The latter may help us understand the reduced inhibitory effect of flecainide in SQT1-hiPSC-CMs. Flecainide did not change the window current of hERG channels. It decelerated the recovery from inactivation in SQT1-hiPSC-CMs and showed no effect on the channel deactivation. These data may help explain why flecainide showed no antiarrhythmic effects in SQT1-hiPSC-CMs and SQT-patients because these effects are different from that of quinidine, ajmaline, ivabradine and mexiletine, which prolonged APD and reduced epinephrine-induced arrhythmic events in SQT1-hiPSC-CMs. Flecainide can inhibit I_Na_, I_Ca-L_, I_to_, I_KATP_, and enhance I_NCX_ and I_K1_ but has no effect on I_Ks_ (Supplementary Table 1) (Watanabe, 2019; Wang et al., 1996; Wang et al., 1993; Follmer and Colatsky, 1990; Caballero et al., 2010; Yunoki et al., 2001). The inhibition of I_to_ and I_KATP_ can prolong APD, which may contribute to the APD-prolongation observed in healthy cardiomyocytes (Table 1) (Borchard and Boisten, 1982; Wang et al., 1993). In the current study, we did not examine the effect of flecainide on these currents. The fact that flecainide failed to prolong APD in SQT1-hiPSC-CMs suggests that its effect on I_to_ and I_KATP_ is probably also attenuated in SQT1-cells.

Ranolazine, an antianginal drug, possesses also antiarrhythmic effects (Frommeyer et al., 2012; Burashnikov et al., 2012). It showed potent antiarrhythmic properties in the rabbit model of pinacidil-induced SQTS (Frommeyer et al., 2016). Ranolazine was shown to block I_Kr_ in dog cardiomyocytes (Antzelevitch et al., 2004a; Schram et al., 2004). In HEK cells expressing WT- and mutant hERG channels, ranolazine inhibited the hERG channel current and its effect was reduced by different mutants including N588K^69^. It shifted the activation curve of WT hERG channels to a more negative potential (Du et al., 2014; Rajamani et al., 2008). It was also shown that ranolazine reduced the time constant of inactivation and showed no effect on the time constant of recovery from inactivation of hERG channels in HEK cells (Rajamani et al., 2008). Effects of ranolazine on gating kinetic parameters of mutant hERG channels have not been shown. Our study analyzed in detail the effects of ranolazine on the activation, inactivation, recovery from inactivation and deactivation of hERG channel currents in both healthy donor and SQT1-hiPSC-CMs. The main findings are that both the activation and inactivation curves were shifted in the same direction and hence the window current was not changed. The time to peak and time constant of deactivation were similar in donor and SQT1-hiPSC-CMs. The time constant of inactivation and time constant of recovery from inactivation were differentially affected by ranolazine in donor- and SQT1-hiPSC-CMs. Besides hERG current, ranolazine can inhibit I_Na_, I_Ca-L_, I_NCX_, I_to_ and I_Ks_ without effect onI_K1_ (Supplementary Table 1) (Antzelevitch et al., 2004b; Qian et al., 2012). Action potential prolongation and shortening induced by ranolazine were observed in wild-type cells (Table 1) (Antzelevitch et al., 2004a; Wu et al., 2004; Frommeyer et al., 2016). The inhibition of I_Ca-L_ can shorten APD, while inhibition of I_Kr_ and I_Ks_ can explain the APD-prolongation. In our SQT1-hiPSC-CMs, ranolazine did not prolong APD, probably, its effect on I_Ks_ was also attenuated or its inhibitory effect on I_Ca-L_ was enhanced, which counteracted the APD-prolonging effect of inhibition of I_Kr_ and I_Ks_.

Taking all the data together, all the examined drugs inhibited the hERG channel current in both donor- and SQT1-hiPSC-CMs. The inhibition by each drug was reduced in SQT1-cells. Though the tested drugs displayed some differences regarding effects on the hERG channel gating kinetics in donor or SQT1-hiPSC-CMs, we observed important features, i.e., 1) quinidine, ajmaline, ivabradine and mexiletine but not amiodarone, flecainide and ranolazine reduced the window current in SQT1-hiPSC-CMs, 2) quinidine, ajmaline, ivabradine and mexiletine but not amiodarone, flecainide and ranolazine increased the time constant of deactivation (the speed of deactivation was reduced). Since quinidine, ajmaline, ivabradine and mexiletine but not amiodarone, flecainide and ranolazine showed APD-prolonging and antiarrhythmic effects in SQT1-hiPSC-CMs (Zhao et al., 2019), we assume that the window current-reducing and deactivation-slowing effect may be important for antiarrhythmic effects of drugs in SQT1-paptient with hERG mutation, at least with a N588K mutation.

Notably, all the seven drugs tested in this study have effects on multiple ion channels (Supplementary Table 1). Theoretically, either an enhancement of inward current or a suppression of outward current or both can prolong APD/QTc. Therefore, the effects of quinidine, ajmaline, ivabradine and mexiletine on ion channel currents other than I_Kr_ may also contribute to their APD/QTc-prolonging and antiarrhythmic effects.

The concentrations of drugs used in the study were higher than that used in patients. The concentrations were selected because they showed noticeable effects (either on I_Kr_ or APD or arrhythmic events) in hiPSC-CMs. The reason for requirement of higher concentration is not clear. Probably, the sensitivity of cells to drugs is reduced due to challenges in cell splitting, single cell isolation or unphysiological cell culture. The high concentration may enhance the toxicity or side effects of a drug and limit the drug application in patients. Besides, cells in other systems express also hERG channels, which may be also inhibited when the drug is used in a SQTS-patient. This may further limit the application of hERG-inhibiting drugs in patients. Therefore, the data from our study cannot be directly translated to clinical use, instead, provide information for designing clinical studies. On the other hand, it is possible that in patients lower concentrations of drugs are required to exert effects same with or similar to that observed in our study. Our hiPSC-CM platform for drug testing can provide possible candidate drugs, but the real efficacy and safe concentrations in patients need to be examined in clinical studies**.**

In summary, we analyzed effects of quinidine, ajmaline, amiodarone, ivabradine, flecainide, mexiletine and ranolazine on hERG channel gating kinetic parameters. We observed that all the tested drugs have some effects on hERG gating parameters in donor or SQT1-hiPSC-CMs (Table 1), but constant effects on hERG window current and deactivation were detected only in the application of quinidine, ajmaline, ivabradine and mexiletine, which may be one of the reasons for their effective antiarrhythmic effects in SQT1-cells.

## Conclusion

From the results we conclude that the window current-reducing and deactivation-slowing effects may be important for the antiarrhythmic effects of drugs in SQT1-paptients with a hERG mutation, at least with a N588K mutation. These findings may help us to search for potential candidate drugs for treating SQT1-patients.

## Study Limitations

We recruited only one SQT1-patient and one healthy donor for this study. Differences among individuals cannot be ruled out. Due to the hurdle to find more SQT1-patients with the same mutation, the study focused on cell line from single patient. Therefore, the study may be relevant for personalized medicine, e.g., for planning a therapeutical strategy for this specific SQT-hiPSC-CM donor.

Isogenic control cells were not used in the study. The possibility that the different gene background in heathy donor and SQT1 cells influenced the drug effects cannot be completely excluded.

Since the concentration of drugs used in the study was higher than that used in patients, possible drug toxicity *in vivo* needs to be considered when interpreting the data of the current study.

All the measurements were performed at room temperature. The possible influence of temperature on drug effects was not examined in this study. In previously reported studies with respect to hERG channel current or drug effect on the current, some were performed at room temperature (Sanguinetti et al., 1995; Trudeau et al., 1995; Snyders and Chaudhary, 1996; Wang et al., 1997; Zhang et al., 1999; Kiesecker et al., 2004; Cordeiro et al., 2005; Rajamani et al., 2008), some at physiological temperature (37°C) (Paul et al., 2002; McPate et al., 2008; Melgari et al., 2015; Melgari et al., 2015). It has been shown that hERG channel current is temperature-sensitive (Zhou et al., 1998; Wang et al., 2003). This may suggest a possible influence of temperature on drug effects. On the other hand, it is also possible that the temperature influence on drug effects is minor because the temperature-sensitivity of the channel does not mean the drug effect is also temperature-sensitive. The temperature may underestimate or overestimate (amplify) drug effects, but it is also possible that temperature does not change drug effect when temperature changes at certain range. Cs^+^ instead of K^+^ was used as the charge carrier for recording hERG current since Cs^+^ can be conducted by hERG channel but not by other K^+^ channels and hence can separate hERG channel current from other K^+^ currents (Schönherr and Heinemann, 1996; Youm et al., 2004; Zhang, 2006). It was reported that Cs^+^ can slow hERG channel inactivation but has no effect on the channel activation (Zhang et al., 2003). This suggests a possibility that Cs^+^ may influence some drug effects. It is possible that Cs^+^ may reduce or enhance drug effects, but it is also possible that it has no influence on some drug effects. Taken together, the drug effects shown in this study were detected under unphysiological conditions and therefore need to be examined in more physiological studies in future.

Another limitation may be the immaturity of hiPSC-CMs. The immature hiPSC-CMs possess similarities but also differences compared with adult human cardiomyocytes. As shown before, the expression profile of some ion channels in hiPSC-CMs may be different from that in adult cardiomyocytes and even more, hiPSC-CMs from different cell lines may also show different expression profile of ion channels (Blinova et al., 2017), which may influence drug effects in hiPSC-CMs. This should be taken into account when interpreting the data.

I_NCX_ is known to have both an inward and an outward component at different phases of APs. Although we reported in Supplementary Table 1 the drug effects on I_NCX_, it is not simple speculating on how its activation/inhibition could affect APD following drug administration. This would require a more detailed analysis that is out of the scope of this manuscript.

## Data Availability

The original contributions presented in the study are included in the article/Supplementary Material, further inquiries can be directed to the corresponding author.
