# Supplementary material for: Effects of Antiarrhythmic Drugs on hERG Gating in Human-Induced Pluripotent Stem Cell-Derived Cardiomyocytes From a Patient With Short QT Syndrome Type 1
Source: Front Pharmacol. 2021 May 7;12:675003. doi: 10.3389/fphar.2021.675003 (PMC8138577; doi:10.3389/fphar.2021.675003)
Supplement: Supplementary file 1 [file DataSheet1.pdf]

Supplementary information

Effects of antiarrhythmic drugs on hERG gating in human-induced pluripotent stem cell-derived cardiomyocytes from a patient with short QT syndrome type 1

Running title: Drug effects on hERG gating

Mengying Huang[1*], Zhenxing Liao[1,2*], Xin Li[1,3*], Zhen Yang[1,2], Xuehui Fan[1,6], Yingrui Li[1], Zhihan Zhao[1], Siegfried Lang[1,4], Lukas Cyganek[4,5], Xiaobo Zhou[1,4,6], Ibrahim Akin[1,4], Martin Borggrefe[1,4], Ibrahim El-Battrawy[1,4]

[1]First Department of Medicine, Faculty of Medicine, University Medical Centre Mannheim (UMM), University of Heidelberg, Mannheim, Germany; [2]North Sichuan Medical College; [3]College of Medical Technology, Chengdu University of Traditional Chinese Medicine; [4]DZHK (German Center for Cardiovascular Research), Partner Sites, Heidelberg-Mannheim and Göttingen, Germany; [5]Stem Cell Unit, Clinic for Cardiology and Pneumology, University Medical Center Göttingen, Göttingen, Germany; [6]Key Laboratory of Medical Electrophysiology of Ministry of Education and Medical Electrophysiological Key Laboratory of Sichuan Province, Institute of Cardiovascular Research, Southwest Medical University, Luzhou, China.

*equally contributed.

Address for correspondence:

Xiaobo Zhou, MD

First Department of Medicine, Faculty of Medicine, University Medical Centre Mannheim, University of Heidelberg, Theodor-Kutzer-Ufer 1-3, 68167 Mannheim, Germany. **E-mail**: Xiaobo.zhou@medma.uni-heidelberg.de

Funding: This study was supported by the Post-doc of German Center for Cardiovascular Research (DZHK) (81Z0500204) and shared expertise (81X2500208).

Conflict of Interest: All authors declared no competing interests for this work.

Material and Methods

Generation of human iPS cells (hiPSC)

Somatic cell isolation and primary culture

Human fibroblast cultures were derived from skin punch biopsies of a healthy donor and patient with SQTS type 1 carrying p.N588K in hERG channel. Skin punch biopsy (3-4 mm) was taken aseptically by a clinical doctor, placed in DMEM (Thermo Fisher Scientific, #11960044) containing 200 U/ml penicillin and 200 µg/ml streptomycin (Thermo Fisher Scientific, #15140122) and transferred to the lab within 48 h. Biopsies were mechanically cut in pieces of 0.5-1 mm, were placed epidermis upside in the cell culture dish and cultured in human fibroblast medium (HFBM) composed of DMEM (Thermo Fisher Scientific, #11960044) supplemented with 10% Fetal Bovine Serum (Thermo Fisher Scientific, #10270106), 1x MEM Non-Essential Amino Acids Solution (Thermo Fisher Scientific, #11140035), 2 mM L-Glutamine (Thermo Fisher Scientific, #25030024), 50 µM β-mercaptoethanol (Serva Electrophoresis, #28625), 10 ng/ml bFGF (Peprotech, #100-18B), 100 U/ml penicillin and 100 µg/ml streptomycin (Thermo Fisher Scientific, #15140122) at 37°C with 5% CO2 atmosphere. Medium was changed every other day. Fibroblasts before passage 3 (p3) was used for generation of iPS cells.

Generation of hiPSCs

HiPSC lines isSTQSa1.7 (GOEi091-A.7, here abbreviated as SQTS1), isSQTSa1.8 (GOEi091-A.8) and isSQTSa1.15 (GOEi091-A.15) were generated from fibroblasts in feeder free culture conditions using the integration-free CytoTune-iPS 2.0 Sendai Reprogramming Kit (Thermo Fisher Scientific, #A16517) with the reprogramming factors OCT4, KLF4, SOX2, c-MYC according to manufacturer's instructions with modifications. In brief, $1.5x10^4$ early passage fibroblasts were plated in two wells of a Matrigel-coated 24-well plate in HFBM two days before transduction. Cells were transduced at 40-50% confluence with Sendai virus cocktail (hKOS: hc-Myc: hKlf4) at a MOI of 10:10:6 according to the counted cell number of extra well (typically $2.5x10^4$ cells/well) in HFBM. Virus was removed after 24 h and HFBM was changed every other day.

HiPSC lines ipWT1.1 (GOEi014-B.1), ipWT1.3 (GOEi014-B.3, here abbreviated as donor) and ipWT1.6 (GOEi014-B.6) were generated from fibroblasts in feeder free culture conditions using the integration-free episomal 4-in-1 CoMiP reprogramming plasmid (Addgene, #63726) with the reprogramming factors OCT4, KLF4, SOX2, c-MYC and short hairpin RNA against p53, as described previously with modifications (1). In brief, $5x10^5$ early passage fibroblasts were used for electroporation with the Nucleofector 2b Device (Lonza) with program P22 or U23 by using the NHDF Nucleofector Kit (Lonza, #VPD-1001) and 2 µg of the reprogramming plasmid. Transfected cells were plated in one well of a Matrigel-coated 6-well plate in HFBM supplemented with 500 µM sodium butyrate (Sigma-Aldrich, #B5887), 2 µM Thiazovivin (Millipore, #420220), 100 U/ml penicillin and 100 µg/ml streptomycin. Medium was changed every other day with HFBM supplemented with 500 µM sodium butyrate.

At day 7 post transduction/transfection, cells were replated in various dilutions in Matrigel-coated 6-well plates in HFBM supplemented with 500 µM sodium butyrate and 2 µM Thiazovivin. From day 8, medium was changed to E8 medium (Thermo Fisher Scientific, #A1517001) supplemented with 500 µM sodium butyrate (day 8-day 11) with daily medium change. Cells were monitored for morphology change and appearance of colonies (typically after 2-3 weeks). Individual colonies with iPSC-like morphology were picked mechanically in Matrigel-coated 12-well plates in E8 medium supplemented with 2 µM Thiazovivin. Newly established iPSC lines were passaged with Versene solution (Thermo Fisher Scientific, #15040066) and cultured in E8 medium supplemented with 2 µM Thiazovivin on the first day after passaging in Matrigel-coated plates for at least ten passages before being used for pluripotency characterization and differentiation experiments.

Characterization of hiPSCs

To examine pluripotent characteristics of generated hiPSCs, cell morphology, alkaline phosphatase activity, expression of endogenous pluripotency markers, Spontaneous differentiation potential were assessed. The hiPSC lines from the donor and STQS1 patient displayed a typical morphology for human pluripotent stem cells and were positive for alkaline phosphatase. In comparison to fibroblasts, generated hiPSC lines showed expression of endogenous pluripotency markers SOX2, OCT4, NANOG, LIN28, FOXD3 and GDF3 at mRNA level proven by RT-PCR. Human

embryonic stem cells (hESCs) were used as positive control, mouse embryonic fibroblasts (MEFs) were used as negative control. The expression pluripotency markers OCT4, SOX2, NANOG, LIN28, SSEA4 and TRA-1-60 was detected in generated iPSC lines. Spontaneous differentiation potential of generated iPSC lines was analysed by embryoid body (EB) formation. Germ layer-specific genes like α-fetoprotein (AFP) and albumin (ALB) (endoderm), cTNT and α-MHC (mesoderm), and tyrosine hydroxylase (TH) and MAP2 (ectoderm) were detected in a developmentally controlled manner during differentiation of EBs (days 0, 8, or 8+25), whereas endogenous OCT4 expression was decreased during spontaneous differentiation. Immunocytochemical staining of spontaneously differentiated hiPSC lines showed expression of endodermal marker AFP, mesodermal-specific α-SMA and ectodermal βIII-tubulin. All data together confirmed the pluripotency and differentiation potential of generated hiPSCs.

Spontaneous in vitro differentiation of hiPSCs

For embryoid body (EB) formation, $5x10^4$ hiPSCs together with $2.5x10^4$ mouse embryonic fibroblasts were plated in each well of a 96-well U-bottom plate in hES medium composed of DMEM-F12 (Thermo Fisher Scientific, #31331028), 15% Knockout Serum Replacement (Thermo Fisher Scientific, #10828028), 1x MEM Non-Essential Amino Acids Solution (Thermo Fisher Scientific, #11140035), 50 µM β-mercaptoethanol (Serva Electrophoresis, #28625) and 2 µM Thiazovivin, the plate was centrifuged at 250 g for 5 min and co-cultures were cultivated in suspension to form multicellular EB aggregates. At day 2, medium was changed to differentiation medium composed of IMDM Glutamax (Thermo Fisher Scientific, #31980022), 20% Fetal Bovine Serum (Thermo Fisher Scientific, #10270106), 1x MEM Non-Essential Amino Acids Solution and 450 µM 1-Thioglycerol (Sigma-Aldrich, #M6145) for further 6 days with medium change every other day. At day 8, EBs were plated onto 0.1% gelatin-coated 6-well plates and cultured for up to one month in differentiation medium with medium change every other day.

Generation of hiPSC-CMs

Frozen aliquots of hiPSCs were thawed and cultured without feeder cells and differentiated into hiPSC-CMs as described with some modifications (2).

Thawing and culture of hiPSCs

To thaw iPS, the following steps were performed.

One day before thawing cells, a T-25 flask coated with Matrigel was put in the incubator.

Mix medium (13 ml E8 medium + 6.5 ul from 10 mM stock solution of Rock inhibitor) was prepared. 2.5ml mix medium was added in the T-25 flask and put into incubator. Frozen hiPS cells were taken out from liquid N2 tank and thawed in hand until only small lumps of ice exist. Then, the cell suspension was transferred to 50 ml Falcon prepared with 5 ml of mixed medium. The cell suspension was centrifuged at 250 x g (1200 rpm), 4min, 20 ° C. The supernatant was discarded and 2.5ml Mix medium was added. After 4x pipetting up and down, the cell suspension was transferred into the T-25 with 2.5 ml mix medium. The T-25 stayed into incubator until the next day.

Differentiation

On the next day, the medium in T-25 flask was changed to E8 medium without ROCK inhibitor. Then the E8 medium was changed every two days. 2 to 4 days later, when cells reached 85-95% confluence, the differentiation was started.

First, cells were treated with EDTA and dissolved in E8 Medium. Then, the cells were counted and plated on Matrigel / Geltrex coated plates (optimal cell density for plating is cell line dependent; 15.000 cell/cm2 - 26.000 cells/cm2 = 150.000-260.000 cells per 6-well). Lastly, E8 medium was added to final volume (3ml per 6-well with 5µM ROCK inhibitor on the first day. The plate was shaken and incubated at 37°C with 5% CO2.

Day -x to -1: Daily medium change with E8 Medium was performed.

Day 0: When cell density reached 85-95% confluence,

the medium was changed to cardiac medium (consisting of RPMI, 1% sodium pyruvate, 1% Pen / Strep, 2% B27 and 200 uM ASC) freshly enforced with 1 uM CHIR ,5 ng / ml BMP4 ,9 ng / ml activin A ,5 ng / ml FGF.

Day 2: After around 48h, the medium changed to cardiac medium freshly enforced with 5 uM IWP4.

Day 4 and later: the medium was changed every 2-3 days to cardiac medium only.

Normally, on day 8 of differentiation some cells start to beat.

Day 13: the cardiac medium was changed to selection medium (consisting of RPMI

without glucose and glutamine ,1% Pen / Strep) freshly added with 440 mM Sodium Lactate Stock and 50 mM 2-mercaptoethanol. The selection medium was changed every day for 4-7 days.

Day 18: the selection medium was changed to cardiac medium (consisting of RPMI, 1% sodium pyruvate, 1% Pen / Strep, 2% B27 and 200 uM ASC) every 2-3 days.

From Day 40: Cells were ready for experiments.

Supplementary Table 1: Summary of drug effects on cardiac ion channel currents

	I_{Na}	$I_{Ca\text{-}L}$	I_{NCX}	I_{to}	I_{Kr}	I_{Ks}	I_{K1}	I_{KATP}
Qui	I[3]	I[4]	N[6] , I[33]	I[4,9]	I[7]	I[8]	N[9], I[80]	I[11]
Ajm	I[12]	I[13]	NA	I[13]	I[14]	NA	I[14]	I[14]
Ami	I[15]	I[5,17]	I[16]	N[17], I[17]	I[17]	I[17]	N[17], I[36]	I[5]
Iva	I[18]	N[18]	NA	NA	I[18]	N[18]	N[19]	NA
Fle	I[20]	N[20], I[35]	A[17]	I[20]	I[21,22]	N[22]	A[23]	I[24]
Mex	I[25]	I[25,27]	I[32]	I[26]	N[22], I[27]	N[22], I[35]	NA	A[30, 31]
Ran	I[28]	I[28]	I[29]	N[28] , I[34]	I[28]	I[28]	N[28]	NA

I_{Na}: peak sodium channel current. $I_{Ca\text{-}L}$: L-type calcium channel current. I_{NCX}: Na/Ca exchanger current. Ito: transient outward potassium channel current. I_{Kr}: rapidly activating delayed rectifier potassium channel current. I_{Ks}: slowly activating delayed rectifier potassium channel current. I_{K1}: inward rectifier potassium channel current. I_{KATP}: ATP-sensitive potassium channel current.

I: inhibition. A: activation. N: no effect. NA: no analysis.

References

1. Diecke S, Lu J, Lee J et al. Novel codon-optimized mini-intronic plasmid for efficient, inexpensive, and xeno-free induction of pluripotency. Scientific reports 2015;5:8081.
2. Tiburcy M, Zimmermann WH. Modeling myocardial growth and hypertrophy in engineered heart muscle. Trends in cardiovascular medicine 2014;24:7-13.
3. Pugsley MK et al. Block of NA+ and K+ currents in rat ventricular myocytes by quinacainol and quinidine. Clin Exp Pharmacol Physiol Jan-Feb 2005;32(1-2):60-5. doi: 10.1111/j.1440-1681.2005.04149.x.
4. Michel D et al. Effects of quinine and quinidine on the transient outward and on the L-type Ca(2+) current in rat ventricular cardiomyocytes. Pharmacology 2002 Aug;65(4):187-92. doi: 10.1159/000064342.
5. Nishida A et al. Inhibition of ATP-sensitive K+ channels and L-type Ca2+ channels by amiodarone elicits contradictory effect on insulin secretion in MIN6 cells. J Pharmacol Sci . 2011;116(1):73-80. doi: 10.1254/jphs.10294fp.
6. Goshima K et al. Inhibition of ouabain-induced increase in Na content of cultured myocardial cells by quinidine and procainamide. J Pharmacol Exp Ther . 1983 Jan;224(1):239-46.
7. Paul AA et al. Inhibition of the current of heterologously expressed HERG potassium channels by flecainide and comparison with quinidine, propafenone and lignocaine. Br J Pharmacol 2002 Jul;136(5):717-29. doi: 10.1038/sj.bjp.0704784.
8. Yang T et al. Probing the mechanisms underlying modulation of quinidine sensitivity to cardiac I(Ks) block by protein kinase A-mediated I(Ks) phosphorylation. Br J Pharmacol 2009 Jul;157(6):952-61. doi: 10.1111/j.1476-5381.2009.00293.x.
9. Imaizumi Y, Giles W R. Quinidine-induced inhibition of transient outward current in cardiac muscle. Am J Physiol . 1987 Sep;253(3 Pt 2):H704-8. doi: 10.1152/ajpheart.1987.253.3.H704.

10. Koepple C et al. Dual Mechanism for Inhibition of Inwardly Rectifying Kir2.x Channels by Quinidine Involving Direct Pore Block and PIP 2-interference. J Pharmacol Exp Ther 2017 May;361(2):209-218. doi: 10.1124/jpet.116.238287.
11. Undrovinas AI et al. Quinidine blocks adenosine 5'-triphosphate-sensitive potassium channels in heart. Am J Physiol 1990 Nov;259(5 Pt 2):H1609-12. doi: 10.1152/ajpheart.1990.259.5.H1609.
12. Khodorov B I, Zaborovskaya L D. Blockade of sodium and potassium channels in the node of Ranvier by ajmaline and N-propyl ajmaline. Gen Physiol Biophys. 1983 Aug;2(4):233-68.
13. Bébarová M et al. Effect of ajmaline on action potential and ionic currents in rat ventricular myocytes. Gen Physiol Biophys 2005 Sep;24(3):311-25.
14. Kiesecker C et al. Class Ia anti-arrhythmic drug ajmaline blocks HERG potassium channels: mode of action. Naunyn Schmiedebergs Arch Pharmacol 2004 Dec;370(6):423-35. doi: 10.1007/s00210-004-0976-8.
15. Suzuki T et al. Atrial selectivity in Na+channel blockade by acute amiodarone. Cardiovasc Res 2013 Apr 1;98(1):136-44. doi: 10.1093/cvr/cvt007.
16. Iwamoto T et al. Na+/Ca2+ exchange inhibitors: a new class of calcium regulators. Cardiovasc Hematol Disord Drug Targets 2007 Sep;7(3):188-98.doi: 10.2174/187152907781745288.
17. Watanabe Y. Cardiac Na +/Ca 2+ exchange stimulators among cardioprotective drugs. J Physiol Sci 2019 Nov;69(6):837-849.doi: 10.1007/s12576-019-00721-5.
18. Haechl N et al. Pharmacological Profile of the Bradycardic Agent Ivabradine on Human Cardiac Ion Channels. Cell Physiol Biochem 2019;53(1):36-48. doi: 10.33594/000000119.
19. Demontis GC et al. Selective Hcn1 channels inhibition by ivabradine in mouse rod photoreceptors. Invest Ophthalmol Vis Sci 2009 Apr;50(4):1948-55. doi: 10.1167/iovs.08-2659.
20. Wang Z et al. Mechanism of flecainide's rate-dependent actions on action potential duration in canine atrial tissue. J Pharmacol Exp Ther 1993 Nov;267(2):575-81.
21. Follmer CH and Colatsky TJ. Block of delayed rectifier potassium current, IK, by flecainide and E-4031 in cat ventricular myocytes. Circulation 1990 Jul;82(1):289-93. doi: 10.1161/01.cir.82.1.289.
22. Wang DW et al. Comparison of the effects of class I anti-arrhythmic drugs, cibenzoline, mexiletine and flecainide, on the delayed rectifier K+ current of guinea-pig ventricular myocytes. J Mol Cell Cardiol 1996 May;28(5):893-903. doi: 10.1006/jmcc.1996.0084.
23. Caballero R et al.Flecainide increases Kir2.1 currents by interacting with cysteine 311, decreasing the polyamine-induced rectification. Proc Natl Acad Sci U S A 2010 Aug 31;107(35):15631-6. doi: 10.1073/pnas.1004021107.
24. Yunoki T et al. The effects of flecainide on ATP-sensitive K(+) channels in pig urethral myocytes. Br J Pharmacol 2001 Jul;133(5):730-8. doi: 10.1038/sj.bjp.0704109.
25. Yonemizu S et al. Inhibitory effects of class I antiarrhythmic agents on Na + and Ca 2+ currents of human iPS cell-derived cardiomyocytes. Regen Ther 2019 Feb 1;10:104-111. doi: 10.1016/j.reth.2018.12.002. eCollection 2019 Jun.
26. Rahm AK et al. Inhibition of cardiac K v 4.3 (I to) channel isoforms by class I antiarrhythmic drugs lidocaine and mexiletine. Eur J Pharmacol 2020 Aug 5;880:173159. doi: 10.1016/j.ejphar.2020.173159. Epub 2020 Apr 29
27. Mitcheson JS and Hancox JC. Modulation by mexiletine of action potentials, L-type Ca current and delayed rectifier K current recorded from isolated rabbit atrioventricular nodal myocytes. Pflugers Arch 1997 Nov;434(6):855-8. doi: 10.1007/s004240050476.
28. Antzelevitch C et al. Electrophysiological effects of ranolazine, a novel antianginal agent with antiarrhythmic properties. Circulation 2004 Aug 24;110(8):904-10.doi: 10.1161/01.CIR.0000139333.83620.5D.
29. Qian C et al. Resveratrol attenuates the Na(+)-dependent intracellular Ca(2+) overload by inhibiting H(2)O(2)-induced increase in late sodium current in ventricular myocytes. PLoS One 2012;7(12):e51358. doi: 10.1371/journal.pone.0051358.

30. Kinoshita H et al. Differential effects of lidocaine and mexiletine on relaxations to ATP-sensitive K+ channel openers in rat aortas. 1999 Apr;90(4):1165-70.doi: 10.1097/00000542-199904000-00032
31. Sato T et al. Mexiletine-induced shortening of the action potential duration of ventricular muscles by activation of ATP-sensitive K+channels. Br J Pharmacol 1995; 115:381-2
32. Demirpençe E et al. Antioxidant action of the antiarrhythmic drug mexiletine in brain membranes. Jpn J Pharmacol.1999 Sep;81(1):7-11. doi: 10.1254/jjp.81.7.
33. Zhang Y H et al. Mode-dependent inhibition by quinidine of Na+-Ca2+ exchanger current from guinea-pig isolated ventricular myocytes. Clin Exp Pharmacol Physiol. 2002 Sep;29(9):777-81. doi: 10.1046/j.1440-1681.2002.03731.x.
34. Hee Jae Kim. Effects of ranolazine on cloned cardiac kv4.3 potassium channels. J Pharmacol Exp Ther. 2011 Dec;339(3):952-8. doi: 10.1124/jpet.111.184176. Epub 2011 Sep 22.
35. Passini E et al. Human In Silico Drug Trials Demonstrate Higher Accuracy than Animal Models in Predicting Clinical Pro-Arrhythmic Cardiotoxicity. Front Physiol. 2017 Sep 12;8:668. doi: 10.3389/fphys.2017.00668. eCollection 2017.
36. Ji Y et al. Class III antiarrhythmic drugs amiodarone and dronedarone impair K IR 2.1 backward trafficking. J Cell Mol Med 2017 Oct;21(10):2514-2523. doi: 10.1111/jcmm.13172.
